# The Duplicated Y-specific *amhy* Gene Is Conserved and Linked to Maleness in Silversides of the Genus *Odontesthes*

**DOI:** 10.3390/genes10090679

**Published:** 2019-09-05

**Authors:** Ricardo S. Hattori, Gustavo M. Somoza, Juan I. Fernandino, Dario C. Colautti, Kaho Miyoshi, Zhuang Gong, Yoji Yamamoto, Carlos A. Strüssmann

**Affiliations:** 1 Unidade de Pesquisa e Desenvolvimento de Campos do Jordão, Sao Paulo Fisheries Institue, APTA/SAA, Campos do Jordão 12460-000, Brazil; 2 Instituto Tecnológico de Chascomús (Consejo Nacional de Investigaciones Científicasy Técnicas—Universidad Nacional de San Martin), Chascomús 7130, Argentina (G.M.S.) (J.I.F.); 3 Instituto de Limnología “Dr. Raúl A. Ringuelet” (ILPLA) (Consejo Nacional de Investigaciones Científicasy Técnicas—Universidad Nacional de La Plata), La Plata 1900, Argentina; 4 Graduate School of Marine Science and Technology, Tokyo University of Marine Science and Technology, 4-5-7 Konan, Minato, Tokyo 108-8477, Japan (K.M.) (Y.Y.); 5 School of Marine Science and Technology, Zhejiang Ocean University, Zhoushan 316002, China

**Keywords:** Temperature-dependent sex determination (TSD), Genotypic sex determination (GSD), pejerrey, sex determination

## Abstract

Sex-determining genes have been successively isolated in several teleosts. In *Odontesthes hatcheri* and *O. bonariensis*, the *amhy* gene has been identified as a master sex-determining gene. However, whether this gene is conserved along related species is still unknown. In this study, the presence of *amhy* and its association with phenotypic sex was analyzed in 10 species of *Odontesthes* genus. The primer sets from *O. hatcheri* that amplify both *amhs* successfully generated fragments that correspond to *amha* and *amhy* in all species. The full sequences of *amhy* and *amha* isolated for four key species revealed higher identity values among presumptive *amhy*, including the 0.5 Kbp insertion in the third intron and *amhy*-specific insertions/deletions. *Amha* was present in all specimens, regardless of species and sex, whereas *amhy* was amplified in most but not all phenotypic males. Complete association between *amhy*-homologue with maleness was found in *O. argentinensis, O. incisa, O. mauleanum, O. perugiae, O. piquava, O. regia,* and *O. smitti,* whereas *O*. *humensis*, *O. mirinensis*, and *O. nigricans* showed varied degrees of phenotypic/genotypic sex mismatch. The conservation of *amhy* gene in *Odontesthes* provide an interesting framework to study the evolution and the ecological interactions of genotypic and environmental sex determination in this group.

## 1. Introduction

The construction of detailed genetic maps while using microsatellites and single nucleotide length polymorphism (SNP) markers, complete genome sequencing, and transcriptome analysis pipelines have promoted the discovery of sex-determining genes in an increasing number of teleost fish. Such findings are uncovering a remarkable variety of master genetic switches of genotypic sex determination in this group of vertebrates [1,2], which is in line with the wide repertoire of reproductive strategies already reported in fish [3]. While there are at least three different sex-determining genes described in *Oryzias* genus [4,5,6], the *sdY* gene, initially characterized in rainbow trout [7], shows a high conservation across Salmonidae family members [8]. The discovery of new sex-determining genes has helped to shed light on the evolutionary mechanisms behind their appearance, which at present include allelic diversification and gene duplication, followed by chromosome transposition [2].

An interesting trend is the increasing number of reports of TGF-beta superfamily members’ involvement in male sex determination. One example is the *amh* gene, a glycoprotein that is expressed by Sertoli cells that encodes for the homologue of the mammalian anti-Müllerian hormone. Although the roles of fish *Amh* have not been fully clarified, it has been associated with the control of germ cell proliferation [9]. In *Odontesthes hatcheri* (Atheriniformes, Atherinopsidae), *amhy* (Y chromosome-linked anti-Müllerian hormone), a male-specific *amh* gene that arises from a duplication of the autosomal *amh* gene *amha* was identified as a male determinant [10]. *amhy* has been found also in the congeneric *O. bonariensis,* the teleost with the strongest thermal sensitivity of sex determination (e.g., temperature-dependent sex determination, or TSD) reported so far [11]. Furthermore, *amhy*-like genes have also been described in the cobaltcap silverside *Hypoatherina tsurugae*, which is an atheriniform from the Atherinidae family [12], the Nile tilapia [13], and more recently in the Northern pike [14]. The receptor of *amh* gene *amhrII* was also described as the sex-determining gene in *Takifugu rubripes* and subsequently in other two congeners [15,16], highlighting the importance of *Amh*-*AmhrII* signaling in sex determination.

The availability of a sex-linked gene in atherinopsids is important, not only to study the evolution of sex determination mechanisms, but also as a molecular tool for assessing environmental impacts on gonadal sex differentiation in wild fish. For example, *amhy* could be used to detect phenotypic/genotypic sex mismatches such as (sex-reversed) XX males or XY females and study their association with unusual environment conditions and/or chemical pollutants with endocrine-disrupting properties. In addition, in *O. bonariensis*, the *amhy* gene can be used to detect YY genotypes, which are expected from mating between sex-reversed XY females and XY males, and that are both viable and fertile in this species [17].

The atherinopsids from *Odontesthes* genus are distributed in the Central and Southern regions of South America and they are mainly estuarine and coastal-marine species but some species inhabit freshwater environments [18]. They comprise important fisheries resources and also present great ecological importance as forage species. Cytogenetic studies revealed a conserved diploid number of 48 chromosomes and the absence of morphologically distinguishable sex chromosomes in three in *Odontesthes* species [10,19,20]. This study investigated whether the *amhy* gene is conserved in 10 species of the *Odontesthes* genus from both the Pacific and Atlantic coasts, as well as some inland habitats, since information on genotypic sex determination of atherinopsids is limited to *O. hatcheri* and *O. bonariensis*. We also characterized the structure of those genes in four key species and examined the genetic association between *amhy* and maleness in wild-caught specimens.

## 2. Materials and Methods

### 2.1. Sample Collection and Species Identification

**Figure 1:**
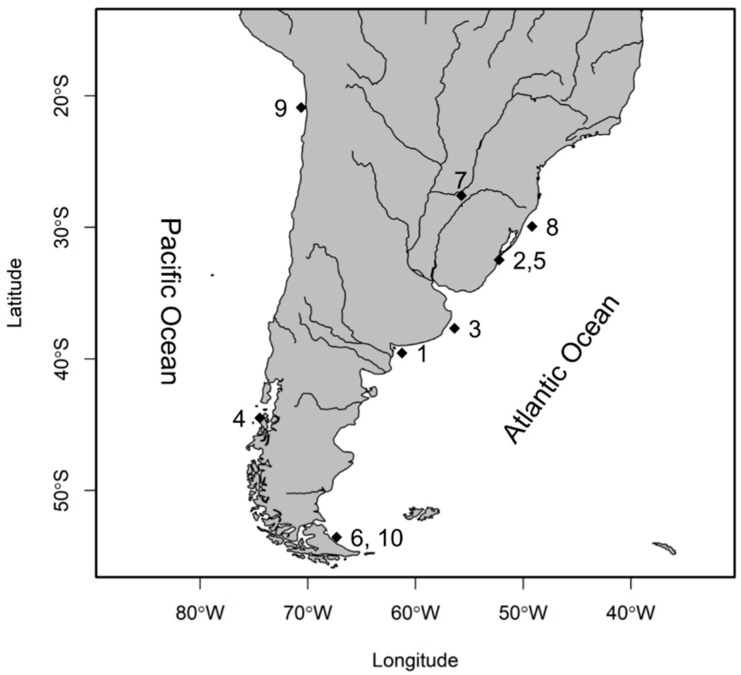
Location of collection sites of *Odontesthes* specimens in South America. 1: *O. argentinensis*, 2: *O. humensis*, 3: *O. incisa*, 4: *O. mauleanum*, 5: *O. mirinensis*, 6: *O. nigricans*, 7: *O. perugiae*, 8: *O. piquava*, 9: *O. regia*, and 10: *O. smitti*.

Materials for this study were collected using gillnets or seine in various localities that are shown in Figure 1 and Table 1. Specimens were identified through morphological and morphometric characters according to Dyer [18] and confirmed by sequence analysis of *cytb* gene in two specimens from each species. The amplification of *cytb* was performed using the primers listed in Table S1, following the procedures described in the next session. Sequences were compared with those that are available in GenBank database. All of the procedures were carried out in accordance with the guidelines for the care and use of animals issued by Sao Paulo Fisheries Institute under the CEEAIP 07/2018, issued on July 2018.

### 2.2. Phenotypic Sex Determination

The majority of the specimens were sexed by direct observation of oocytes or milt upon application of abdominal pressure or by macroscopic inspection of the gonads. In *O. perugiae*, gonads were processed by histological analysis. For this purpose, fish were fixed in Bouinʼs solution overnight and then preserved in 70% ethanol. The trunk region that contained the gonad was embedded in paraffin and transversally sectioned at a thickness of 6 μm. Sections were stained with hematoxylin-eosin and examined under a microscope while using criteria described previously [10].

### 2.3. DNA Extraction and Sex Genotyping by amhy Amplification

Total DNA extraction from caudal fin or muscle and PCR using primer sets described in Table S1 were performed following our previous study [10]. The amplification conditions for both primer sets consisted of 3 min at 94 °C, followed by 35 cycles of 30 s at 94 °C, 30 s at 60 °C, and 2 min at 72 °C, and a final elongation for 5 min at 72 °C. PCR products were electrophoresed in 1% agarose gel and stained with ethidium bromide or SYBR^®^ Safe DNA gel stain (Invitrogen, Carlsbad, CA, USA). Samples without *amhy* amplification (*amhy*^-^) were denoted as XX, whereas those with *amhy* amplification (*amhy*^+^) were scored as XY/YY, because this assay does not distinguish hetero- from homozygotes. Two *amhy*-positive individuals each from samples of *O. argentinensis*, *O. incisa*, *O. regia*, and *O. smitti* were selected for the complete sequencing of *amhy* and *amha* genes, with the purpose of assessing its conservation in terms of *Odontesthes* species taxonomy and also gene structure. These four species, in addition to *O. hatcheri* and *O. bonariensis*, were chosen as to represent the main taxonomic clades of *Odontesthes* [21]. Fragments were excised from the gel, purified while using illustra^TM^ GFX PCR DNA and Gel Band Purification kit (GE Healthcare Life Sciences, Buckinghamshire, UK), labeled with Big Dye Terminator v3.1 Cycle Sequencing Kit (ABI, Foster City, CA, USA), and sequenced in both directions by the ABI PRISM® 3130XL capillary sequencer using primers from Table S1. The sequences were analyzed using GENETYX v. 11 (Genetyx Corporation, Tokyo, Japan) and then deposited on GenBank (NCBI accession codes: #MN380791; #MN380792, #MN380793, #MN380794, #MN380795, #MN380796, #MN380797, and #MN380798).

## 3. Results

*Amha* was present in all specimens, regardless of species and sex, whereas *amhy* was amplified in most but not all phenotypic males. The full sequence of *amhy* isolated for four key species revealed the characteristic features of *O. hatcheri amhy*. Complete association between *amhy*-homologue with maleness was found in all species, except in *O*. *humensis*, *O. mirinensis*, and *O. nigricans*.

### 3.1. Amplification and Sequencing of amhy and amha Genes

**Figure 2:**
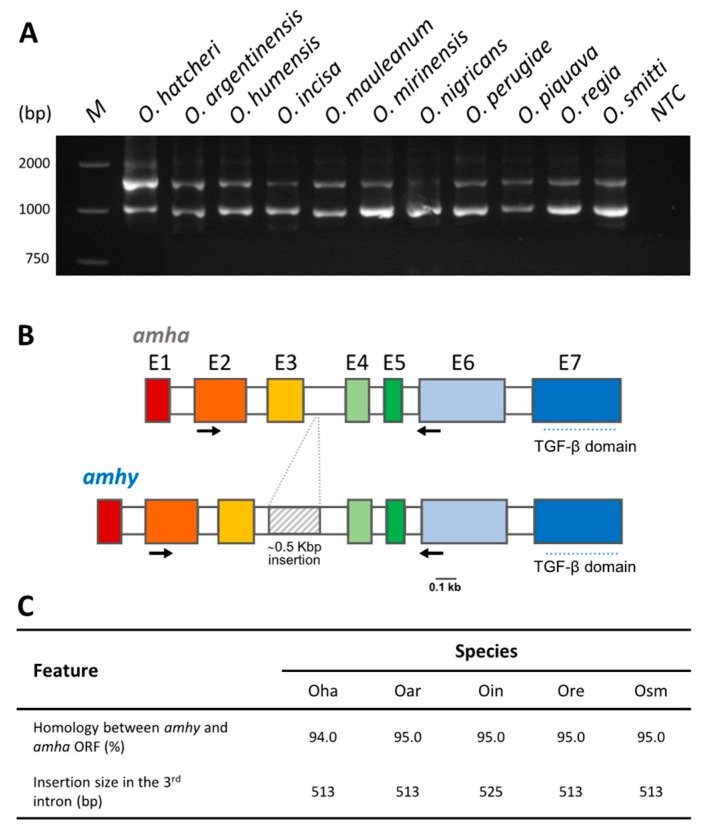
*amhy* gene amplification pattern, gene structure, and nucleotide sequence comparison *Odontesthes* species. (**A**) Pattern of *amhy* (upper band) and *amha* (lower band) amplification using primers flanking the third intron in presumable genotypic males. M: molecular weight marker; NTC: non-template control. (**B**) Schematic representation of *amhy* and *amha* gene structure in the four key *Odontesthes* species. Exons (E1 to E7) are represented by colored boxes and introns by open boxes; corresponding exons of each *locus* are represented by the same color. The gray box represents the insertion in the third intron. Arrows indicate the position of primers used in (**A**). (**C**) Nucleotide identity comparison of predicted *amha* and *amhy* Open reading frames and the size of insertion in the third intron. Oha: *Odontesthes hatcheri*; Oar: *O. argentinensis;* Oin: *O. incisa;* Ore: *O. regia;* Osm: *O. smitti*.

**Figure 3:**
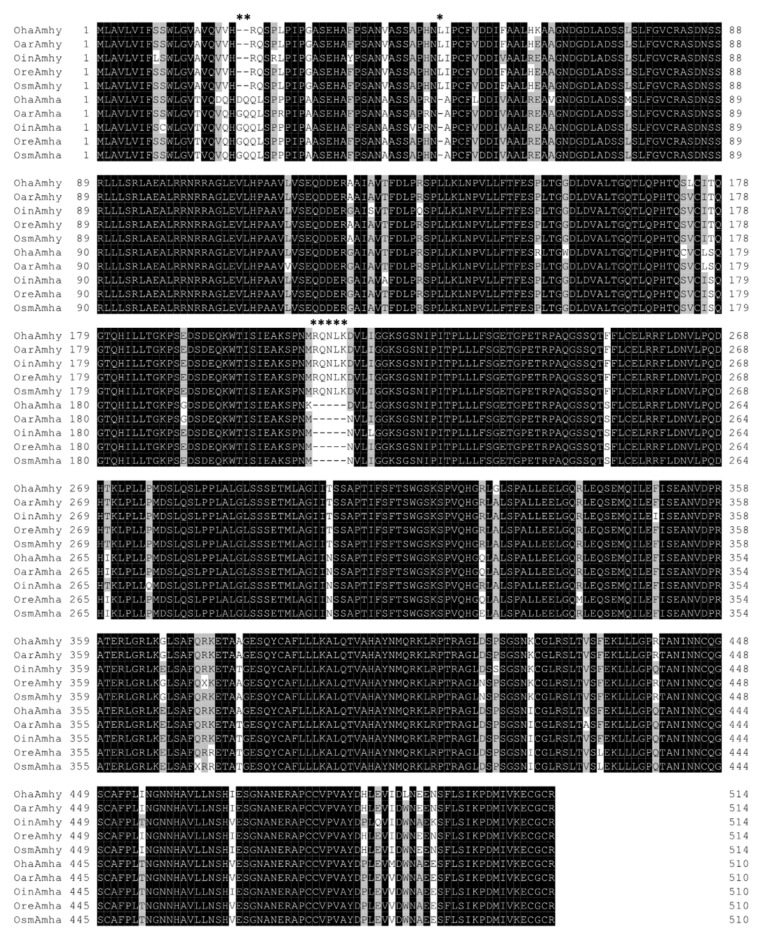
Alignment of the predicted amino acid sequences of *amha* and *amhy* genes in four *Odontesthes* species. Oha: *Odontesthes hatcheri*; Oar: *O. argentinensis;* Oin: *O. incisa;* Ore: *O. regia;* Osm: *O. smitti*. Identical residues and conserved substitutions are highlighted by black and gray background, respectively. Asterisks represent conserved insertions and deletion. The boxed region represents the TGF-beta domain with conserved seven canonical cystein residues (S).

PCR using the primer set that amplifies both *amha* and *amhy* genes in *O. hatcheri* generally resulted in two bands in males and one in females of most of the specimens examined, except in *O. humensis*, *O. mirinensis*, and *O. nigricans* (see details of genotype/phenotype agreement in the next section). The approximate band sizes did not differ from those of *O. hatcheri* (Figure 2A; the pattern of two bands is shown), which have 1614 bp and 1057 bp, respectively, for *amhy* and *amha*. Sequence analysis of *amhy* and *amha* in four species (*O. argentinensis*, *O. incisa*, *O. regia*, and *O. smitti*) revealed the presence of the characteristic insertion in the third intron of *amhy* homologue (Figure 2B), which was slightly larger in *O. incisa* (525 bp) than in other species (513 bp) (Figure 2C). The nucleotide identity between the open reading frame (ORF) of both genes was 95% in all four species (Figure 2C). The alignment of predicted amino acid (aa) sequence with *amhy* of *O. hatcheri,* and *O. bonariensis* showed the conserved insertion and deletion in the N-terminal region of protein, which are not present in the *amha* homologue: a 2 and 5 amino acid insertion in the position +20 and +111, respectively, and a 1 amino acid deletion in the position +47 (Figure 3). The N-terminal was shown to be a more variable region than the C-terminal domain.

### 3.2. Correlation Between the Presence of amhy and Phenotypic Sex 

Mismatches between the genotypic sex (XY/YY or XX), inferred from the presence or absence of *amhy*, respectively, and the phenotypic sex were identified in three species (Table 2). *Odontesthes mirinensis* had the highest mismatch (sex reversal) rate (38.1%), followed by *O. nigricans* (33.3%) and *O. humensis* (25%). In the three species, the percentages of female-to-male sex reversal were higher than those of male-to-female (Table 2).

## 4. Discussion

The silversides (Atheriniformes) stand out among the group of teleosts with temperature-dependent sex determination, because of the high sensitiveness of their gonads to both high (masculinizing) and low (feminizing) temperatures during early life [22,23]. The discovery of the testis-determining gene *amhy* in some of these species has provided a valuable tool in the research on interactions between sex determination mechanisms in this group of fishes [11,12,17]. The presence of *amhy* orthologue was investigated in South American silversides in order to increase the usefulness of silversides on sex determination research and to try to trace back the evolution of *amhy*.

Homologues of *amhy* and *amha* genes of *O. hatcheri* [10] were successfully found in all of the *Odontesthes* species examined. The *amhy* showed a high genetic association with maleness in most species, with the exception of three species, in which mismatches between genotypic and phenotypic sex were relatively frequent and supposedly caused by sex reversal due to environmental conditions [23] (see discussion below). Sequence analyses of *amhy* coding and non-coding regions in the four selected species confirmed the characteristics indels in the N-terminal region of the protein and the insertion of about 0.5 Kbp in the third intron, as also found in *O. bonariensis* [11], which suggested a conserved structure of *amhy* in *Odontesthes* species. Although the third intron insertion was slightly longer in *O. incisa*, other introns and also the TGF-beta domain also showed a high conservation in terms of size and nucleotide sequence between the four species. These results, together with high genetic association of *amhy* with maleness in most of the species, suggest that *amhy* is likely a functional and conserved sex-determining gene in most, if not all, *Odontesthes* species.

The *Odontesthes* genus comprises over 16 species described and recent phylogenetic analyses by mitochondrial and nuclear genes have proposed the *O. smitti* and *O. nigricans* as the most plesiomorphic species [21]. The presence of *amhy* in this basal group supports the appearance of *amhy* at least before or during the rise of *Odontesthes* genus, which is estimated between around 5 and 20 million of years ago [21]. The search for *amhy* or alternative sex-determining genes has now been expanded to include other Atheriniforms, such as atherinopsids of the *Basilichthys* genus, sister genus of *Odontesthes*, North American atherinopsids, and atherinids. A constraint for the identification of sex-determining genes lies on their higher substitution rates when compared to the respective paralogues, based on comparative analysis on *dmy*, *DM-W*, and *SRY* genes [24]. For example, a duplicated *amh* that was highly linked to maleness has been recently identified in an Old World Atheriniform (Atherinidae), the cobaltcap silverside *Hypoatherina tsurugae* [12]. However, the *amhy*-like gene in this species has surprisingly lost two exons during its evolution, thus acquiring a truncated gene structure. For that reason, it is still uncertain whether the cobaltcap “*amhy*” is orthologous to the *amhy* that was identified in *Odontesthes* genus. Another constraint is related to the high sensitivity of sex to environmental conditions in many of these members [25,26,27,28], which can give rise to mismatch between genotypic sex and phenotypic sex in the wild. Nevertheless, the discovery of *amhy* or alternative sex-determining genes can help to shed light on the evolution and interaction of both genotypic and environmental sex determination in atheriniforms.

The analysis of genotypic sex based on sex-determining gene presence/absence evidenced the occurrence of sex mismatches in several species. A comparative analysis between species that takes the type of habitat into consideration points to a trend of higher sex reversal rates in inland waters as compared to marine or estuarine environments. Sex reversal occurred in both directions in *O. humensis* and *O. mirinensis*, with XY/YY females (feminization) and XX males (masculinization), similarly to the reported in *O. bonariensis* [11,17], which also inhabits shallow lakes. It is still unknown if such sex-reversed animals are formed naturally as part of the species reproductive strategy or if they are the consequence of changes in natural environments, such as global climate change. For instance, environmentally relevant low and high temperatures effectively feminize and masculinize many silversides, respectively [23]. It must be pointed out that, although the current scenario of global climate change points to a gradual increase in water and air temperature, climatic events with unusually low temperatures have also been recorded rather frequently [29]. Alternatively, the release of endocrine-disrupting chemicals with androgenic or estrogenic effect or alterations in habitats due to anthropogenic activities can also account for sex reversal in the wild [30,31,32]. The morphology of lakes in the South-Eastern region of South America (shallow and small volume) make them highly vulnerable to environmental changes. On the other hand, marine and estuarine regions have *per se* a higher buffer potential, and therefore comprise environments that are more resilient to alterations in the physical and chemical factors. Nevertheless, periodic field surveys in various types of environments in combination with tools for recording the temperature experienced by fish during the early life stages can contribute to strengthening the correlations between phenotype and environment.

Overall, the *amhy* gene comprises a biomarker of genetic sex with a wide usefulness that ranged from evolution studies of sex-determining mechanism in fish to ecological studies, like the direct monitoring of natural populations [17,33]. According to the status of these wild populations, *amhy* could also be used for restocking programs, whereby distortions in the sex genotype/phenotype could be mitigated by releases of seeds with the appropriate combination of genotypes and phenotypes. The identification of *amhy* in other groups may increase the potential of silversides in studies regarding the assessment of climate change impacts on fish sex determination and population structure.

## 5. Conclusions

In conclusion, this study showed that the *amhy* gene is structurally conserved and has a high genetic association with maleness along the main clades of the genus *Odontesthes*. The presence of a genotypic sex determinant provides a useful tool for studies on the evolution of and the ecological interactions between genotypic and environmental sex determination in this group.

## Figures and Tables

**Table 1:** Information on the location and type of environment of the collection sites, number and sex of specimens analyzed in this study.

Species	Sampling Locality/Origin	Environment	Number of Samples (F + M + ND)
*O. argentinensis*	Los Pocitos, San Blás (ARG)	Marine	10 (6 + 4 + 0)
*O. humensis*	Mirin Lagoon, Taim (BRA)	Inland water	20 (4 + 14 + 2)
*O. incisa*	Mar del Plata (ARG)	Marine	35 (26 + 9+ 0)
*O. mauleanum*	Valdivia (CHI)	Estuarine	24 (8 + 14 + 2)
*O. mirinensis*	Mirin Lagoon, Taim (BRA)	Inland water	21 (7 + 14 + 0)
*O. nigricans*	Punta María (ARG)	Marine	12 (7 + 5 + 0)
*O. perugiae*	Cambe Cué Lagoon, Corrientes (ARG)	Inland water	16 (0 + 0 + 16)
	Paraná River, Encarnación (PAR)	Inland water	10 (3 + 7 + 0)
*O. piquava*	Tramandaí Lagoon, Tramandaí (BRA)	Estuarine	21 (13 + 7 + 1)
*O. regia*	Playa Blanca, Iquique (CHI)	Marine	36 (31 + 5 + 0)
*O. smitti*	Mar del Plata (ARG)	Marine	4 (2 + 2 + 0)
	Punta María (ARG)	Marine	12 (6 + 6 + 0)
Total			246 (114 + 110 + 22)

ARG: Argentina; BRA: Brazil; PAR: Paraguay; CHI: Chile; F: female; M: male; ND: not determined.

**Table 2:** Correlation between genotypic and phenotypic sex in *Odontesthes* species.

*Species* (Population)	*amhy*^+^ (XY/YY)		Sex Reversal (%)	*amhy*^–^ (XX)		Sex Reversal (%)
	F	M		F	M	
*O. argentinensis*	0	4	0	6	0	0
*O. humensis*	1	12	1 (7.7)	2	1	1 (33.3)
O. incisa *	0	6	0	21	0	0
*O. mauleanum*	0	3	0	5	0	0
*O. mirinensis*	3	9	3 (33.3)	4	5	5 (55.6)
*O. nigricans*	0	2	0	6	4	4 (40)
*O. perugiae* (Paraná River pop.)	0	7	0	3	0	0
O. piquava *	0	2	0	13	0	0
O. regia *	0	5	0	31	0	0
*O. smitti* (Mar del Plata pop.)	0	2	0	2	0	0
(Punta Maria pop.)	0	6	0	6	0	0

*amhy*^+^: samples with *amhy* amplification; *amhy*^–^: samples without *amhy* amplification. Asterisks (*) after the species name indicate that phenotypic sex ratios differ significantly from 1:1. Pop.: population; F: female; M: male.

## Supplementary Materials

The following are available online at https://www.mdpi.com/2073-4425/10/9/679/s1, Table S1: List of primers used to amplify and sequence the *cytb* gene and the homologues of *amha* and *amhy.*
