# Supplementary material for: The Duplicated Y-specific amhy Gene Is Conserved and Linked to Maleness in Silversides of the Genus Odontesthes
Source: Genes (Basel). 2019 Sep 5;10(9):679. doi: 10.3390/genes10090679 (PMC6770987; doi:10.3390/genes10090679)
Supplement: Supplementary file 1 [file genes-10-00679-s001.pdf]

Supplemental Material

Table S1. List of primers used to amplify and sequence the *cytb* gene and the homologues of *amha* and *amhy.*

Purpose	Oligo name	Oligo sequence ('5 – '3)
cytb	cytbfw	ACCGTTGTATTCAACTACAAGAACC
amplification	cytbrv	GCTACTAGTGCATTGTCATTCGAG
amha	Amha Fw	ACGCGGGTCACACAGGCGTTTC
amplification	Amha Rv	TAACTAGTCATACTTTTCATTTAATCTATAAAG
		ATTCAGAATGCTACA
amhy	Amhy Fw	GCACGTCGGAGGTCGGAGTTTC
amplification	Amhy Rv	TGACACTCAGCAAGGGTTACGGCAAA
amhy and	Amh Fw1	GACCTCCCTCGGTCCCCTTT
amha	Amh Fw2	GAGGCAAACGTGGATCCCAG
Sequencing	Amh Rv1	GTCACCGCCTGTGAGGGGACTTTCAAAGGTGA
	Amh Rv2	GTGCACACGACATATGACCATGA
	Amh Rv3	GTCTCGCTGGAGGATAAGCCAA
	Amh Rv4	CTGCATGTTATATGCATGGGCC
